# Mitochondrial regulatory mechanisms in spinal cord injury: A narrative review

**DOI:** 10.1097/MD.0000000000031930

**Published:** 2022-11-18

**Authors:** Chengjiang Liu, Yidong Liu, Boyuan Ma, Mengmeng Zhou, Xinyan Zhao, Xuanhao Fu, Shunli Kan, Wei Hu, Rusen Zhu

**Affiliations:** a Department of Spine Surgery, Tianjin Union Medical Center Tianjin, Tianjin, China.

**Keywords:** cuproptosis, mitochondrial, mitophagy, spinal cord injury, therapeutic

## Abstract

Spinal cord injury is a severe central nervous system injury that results in the permanent loss of motor, sensory, and autonomic functions below the level of injury with limited recovery. The pathological process of spinal cord injury includes primary and secondary injuries, characterized by a progressive cascade. Secondary injury impairs the ability of the mitochondria to maintain homeostasis and leads to calcium overload, excitotoxicity, and oxidative stress, further exacerbating the injury. The defective mitochondrial function observed in these pathologies accelerates neuronal cell death and inhibits regeneration. Treatment of spinal cord injury by preserving mitochondrial biological function is a promising, although still underexplored, therapeutic strategy. This review aimed to explore mitochondrial-based therapeutic advances after spinal cord injury. Specifically, it briefly describes the characteristics of spinal cord injury. It then broadly discusses the drugs used to protect the mitochondria (e.g., cyclosporine A, acetyl-L-carnitine, and alpha-tocopherol), phenomena associated with mitochondrial damage processes (e.g., mitophagy, ferroptosis, and cuproptosis), mitochondrial transplantation for nerve cell regeneration, and innovative mitochondrial combined protection therapy.

## 1. Introduction

The pathological process of spinal cord injury (SCI) includes primary and secondary injuries, characterized by a progressive cascade.^[1–3]^ Primary injuries are immediate, generally confined to the injury site, and are irreversible. In secondary injury, vasoconstriction and reduced oxygen supply caused by vascular rupture lead to tissue ischemia and hypoxia, which directly reduces the function of mitochondria to maintain homeostasis,^[4]^ resulting in the loss of adenosine triphosphate (ATP)-dependent cellular functions and the emergence of calcium ions. Secondary pathological reactions such as overload, oxidative stress, and excitatory amino acid toxicity further exacerbate the damage.^[5]^ In the secondary injury process that continues to progress from hours to days, timely and effective intervention can reverse the injury to a certain extent.^[6]^ Therefore, early and effective intervention of secondary injury is key to repair spinal cord injury.^[7]^ It is a promising therapeutic measure that reduces mitochondrial damage to maximize the preservation of neural function.^[8]^

This review discusses changes in mitochondrial biological function in spinal cord injury and the homeostasis imbalance it induces and outlines promising therapeutic targets and approaches.

## 2. Pathological changes of mitochondria after spinal cord injury

Mitochondria are double-membrane organelles that supply ATP for metabolism through oxidative phosphorylation via the electron transport chain (ETC). The mitochondrial outer membrane contains voltage-dependent anion channels that allow the passage of small molecules such as anions and ATP.^[9–11]^ The function of the inner membrane, which controls ATP production by regulating the ETC, is more complex. In oxidative respiration, the asymmetric distribution of ion concentration^[12]^ on both sides of the plasma membrane maintains the electrochemical gradient necessary for ATP production, that is, the mitochondrial membrane potential.^[13]^ When mitochondria are damaged, ETC function is disordered, mitochondrial membrane potential disappears, and ATP synthesis is blocked. At the same time, electrons leak into the mitochondrial matrix, combine with O_2_, and form various reactive oxygen species (reactive oxygen species [ROS]).^[14,15]^ Cell death is triggered when ROS accumulation exceeds the scavenging capacity of the endogenous antioxidant system. In addition, neurons have minimal buffering capacity against oxidative stress^[16,17]^ and even mild mitochondrial damage can lead to severe functional impairment.^[18,19]^

With the deepening of research, researchers have gradually realized that the mitochondria in the central nervous system is not only an energy supplier but also a key factor affecting neuronal function and metabolic homeostasis.^[20,21]^ This is reflected in the following aspects.

### 2.1. Ca^2+^ imbalance and mitochondrial permeability transition pore (mPTP) opening

Ca^2+^ plays a wide range of roles in signaling, but overloading can lead to cellular damage. After spinal cord injury, the mitochondrial membrane is depolarized, and neurotransmitters such as glutamate are released in large quantities, which bind to NMDA-type receptors on the cell membrane to form Ca^2+^ hyperpermeable channels, resulting in increased Ca^2+^ influx.^[19,22]^ Ca^2+^ is taken up by the Na^+^-Ca^2+^ exchanger on the mitochondrial surface, which activates a variety of proteolytic enzymes and lipid lysins, leading to plasma membrane damage and reduced ATP production. At the same time, inhibition of the Ca^2+^ exchanger function further increases the accumulation of Ca^2+^, forming a vicious circle, eventually leading to skeletal proteolysis.^[23–26]^

The mPTP spans the inner and outer mitochondrial membranes and comprises multiple protein dynamics. Its main backbone is a voltage-dependent anion channel located in the outer membrane and adenine nucleotide translocons located in the inner membrane.^[27]^ Under physiological conditions, mPTP can release Ca^2+^ in the mitochondrial matrix by transient opening to maintain ion balance. However, when the accumulation of Ca^2+^ in the matrix exceeds a certain threshold, it triggers the continuous opening of the mPTP, resulting in the disappearance of the mitochondrial inner membrane potential, cessation of ATP synthesis, entry of water molecules into the mitochondria, swelling of the matrix, and rupture of the outer membrane.^[27–29]^ Subsequently, a large amount of Ca^2+^, ROS, and pro-apoptotic proteins are released into the cytoplasm, which activates DNase- and Caspase-3-mediated downstream apoptotic pathways in the nucleus, resulting in cytoskeletal proteolysis.^[30,31]^ In addition, the threshold of calcium ion concentration required to trigger the sustained opening of the mPTP is lower in spinal cord mitochondria than in brain mitochondria, suggesting that mitochondrial dysfunction plays a vital role in the sustained loss of neurons during spinal cord injury.^[32,33]^

### 2.2. Oxidative stress and lipid peroxidation (LP) caused by mitochondrial damage

**Figure 1.**
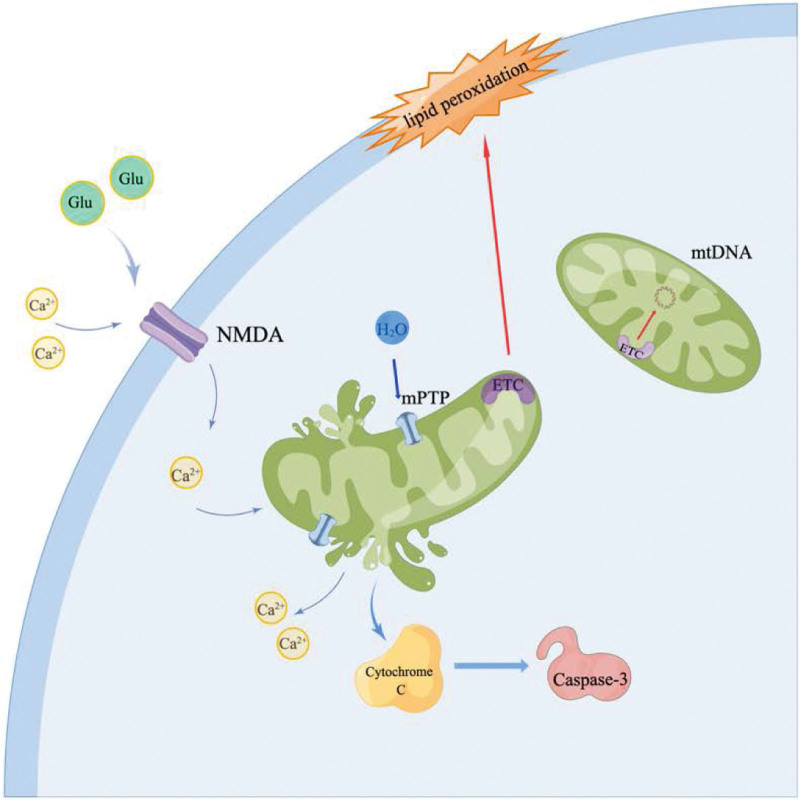
Glutamate is released extracellularly after mechanical injury and activates cellular NMDA-type receptors. Ca^2+^ enters the cell and is taken up by the mitochondria, causing persistent opening of the mPTP. Excess water molecules enter the mitochondria through the mPTP, causing rupture. The release of Ca^2+^, cytochrome c, and other factors activate Caspase-3-dependent or independent apoptosis. mPTP = mitochondrial permeability transition pore.

Mitochondria are considered a source of LP and targets of oxidative stress. Oxidative stress in most neural cells is initiated by mitochondrial production of reactive peroxynitrite (PN).^[34,35]^ Glutamate released by mechanical injury increases Ca^2+^ influx, activating mitochondrial nitric oxide synthase (mtNOS) to generate nitric oxide (NO·). At the same time, the electrons leak to form superoxide radicals (O_2_-·), and the 2 react rapidly to form peroxynitrite anions (ONOO-).^[36,37]^ A large amount of ONOO- traps H + to form peroxynitrite (ONOOH), which is then decomposed into nitrogen dioxide (NO_2_·) and hydroxyl radicals (·OH) with high oxidative activity. The PN anion can react with carbon dioxide (CO_2_) to form nitrosoperoxycarbonate (ONOOCO_2_-), which decomposes to form NO_2_· and carbonate (CO_3_-) radicals.^[38]^ The propylene group in the polyunsaturated fatty acid on the plasma membrane has a double bond structure such that 1 electron in the carbon-hydrogen bond is pulled to 1 side, and the distance between the paired electrons increases, so it is vulnerable to the attack of reactive oxygen molecules, and active lipids (L·) are produced.^[39]^ L· can react with O_2_ to form LP free radicals (LOO·), acquire adjacent H, generate LOOH and a second L·, initiate a chain reaction, and destroy the integrity of the plasma membrane.^[27]^ The end products of LP, such as 4-hydroxynonenal and acrolein, bind to essential amino acids, alter their structure and function, and are neurotoxic.^[40]^ When LP progresses within mitochondria, it results in the loss of membrane potential, affecting ATP production, respiratory failure, and ultimately mPTP opening, triggering apoptosis.^[41]^ Furthermore, since PN has a half-life of approximately 1 second, it has sufficient time to leave the mitochondria, damaging other cellular components.^[29]^ In addition, studies have shown that endoplasmic reticulum stress occurs in astrocytes after ischemic injury, resulting in calcium leakage. It interferes with the mitochondrial membrane and respiratory function and activates the NF-κB pathway, which mediates inflammation. The tissue damage was further exacerbated at this point^[42–44]^ (Fig. 1).

### 2.3. Mitochondrial DNA (MtDNA) damage leads to dysfunction

The mitochondrial matrix contains circular MtDNA, which encodes various respiratory chain subunits and is essential for cellular oxidative phosphorylation.^[39]^ MtDNA can also promote energy production through extensive interactions with nuclear genes.^[45–49]^ Positionally, MtDNA is close to the ETC, is vulnerable to oxidative molecules, lacks protection and repair means like nuclear DNA, has a high risk of damage, and has a relatively high mutation frequency.^[50,51]^ When MtDNA is mutated and cytochrome oxidase is absent, severe respiratory chain and proton pump dysfunction often occurs, and the H + concentration gradient disappears.^[52–56]^ In addition, MtDNA lacks histones and has a very loose structure, which is prone to breakage after being bound by ROS, thereby affecting the synthesis and function of respiratory enzymes on the mitochondrial membrane.^[57]^ Currently, mtDNA deletions have been identified in various neurodegenerative diseases. Therefore, it can be speculated that MtDNA damage exacerbates mitochondrial dysfunction and further damages the local tissue of the spinal cord injury.^[58,59]^

### 2.4. Mitochondria promote axon regeneration

After spinal cord injury, axons demyelinate and lose their nerve conduction function. Mitochondria play an essential role in the repair, regeneration, elongation, and branching of axons following injury.^[60,61]^ Axonal regeneration occurs distally, forming peripheral (P-area), central (C-area), and growth cones. The P region is mainly composed of actin filaments and requires ATP assembly to support pseudopodia protrusions and control the rate of axon regeneration. Tubulin is concentrated in the C region and probes the intracellular environment via highly dynamic depolymerization/polymerization at tip.^[62]^ In addition, microtubules are the action tracks of kinesin and motor proteins, which can target and bind to mitochondria so that they are transported along axons and ensure the supply of ATP. Axon regeneration requires the consumption of 50% of ATP in the cell and is, therefore, highly dependent on the integrity of mitochondrial function. Studies have shown that most mitochondria are distributed along the axon after spinal cord injury, in a state of stagnation, and axon regeneration is slowed down.^[62,63]^ Reducing the expression of syntaphilin, a mitochondrial outer membrane protein, significantly reduced the proportion of stalled mitochondria. In a Ca^2+^ environment, syntaphilin can bind to kinesin-1, limiting its ability to bind mitochondria, thereby increasing the proportion of stalled mitochondria.^[64]^ Therefore, Ca^2+^ distribution along axons after spinal cord injury may be one of the reasons for the inhibition of axon regeneration.^[65–67]^ In addition, microtubule-associated protein is thought to positively affect the mitochondrial transport. It can act as an intermediate ligand to improve the stability of molecular motors when transporting mitochondria, thereby promoting axonal regeneration after spinal cord injury.^[68]^

### 2.5. Mitophagy dysregulation

Mitochondrial dysfunction is implicated in the initiation of neuronal damage during spinal cord ischemia. It is also critical for the cascade of secondary damage and subsequent damage to the mitochondria and neuronal cell death.^[69–71]^ The removal of damaged mitochondria by autophagy is known as mitophagy. Mitophagy plays a beneficial role during the ischemia-reperfusion phase after spinal cord injury. Inhibition of autophagy by drugs or genes rescues ischemic neurons.^[72]^ Mitophagy is critical for the regulation of mitochondrial homeostasis and promoting of cell survival.^[73]^ Evidence suggests that PINK1/Parkin is a canonical mitophagy pathway.^[74–78]^ PINK1 targets the recruitment of parkin to damaged mitochondria, which ubiquitinates several target proteins on the mitochondrial outer membrane and induces autophagy and degradation.^[79]^ A series of in vivo and in vitro experiments have shown that salidroside can enhance the PINK1-Parkin signaling pathway and promote mitophagy.^[80]^ In Mao’s study, maltol administration blocked OS signaling and apoptosis-mediated neuronal cell death after SCI by triggering the expression of Nrf2. At the same time, the authors found that maltol treatment enhanced PINK1/Parkin-mediated mitophagy in PC12 cells and promoted the recovery of mitochondrial function.^[81]^ Therefore, when mitochondria are damaged, PINK1 can recruit Parkin by selectively accumulating in damaged mitochondria, inducing the degradation of damaged mitochondria and reducing neuronal cell damage after SCI.

## 3. Mitochondrial-based treatment of spinal cord injury

Pathological changes after spinal cord injury were divided into 2 stages. Severe primary injury results in complete spinal cord transection, and pharmacological intervention is suboptimal due to the lack of residual tissue with repair potential.^[82]^ Secondary injuries can last for weeks or months and often have more severe consequences than primary injuries, including neuronal apoptosis, progressive demyelination, inflammation, and glial scarring,^[83,84]^ leading to progressive damage. At present, cell transplantation combined with drug therapy is often used for secondary injury, but the therapeutic effect is not satisfactory due to many problems, such as the killing of transplanted cells by local inflammation and insufficient cell migration ability.^[85,86]^ Since mitochondria are the initiating “devices” of various secondary damages, better therapeutic effects can only be achieved by restoring mitochondrial function within a specific time window.^[87–91]^

### 3.1. Uncoupling agent

Uncouplers of the mitochondrial electron transport system uncouple the ETC from complex V, allowing hydrogen atoms to diffuse from the inner and outer mitochondrial intermembrane spaces to the matrix, eliminating the hydrogen ion gradient. The electrons pass through the complex without ADP phosphorylation, which increases the transport rate, helps maintain the mitochondrial inner membrane potential, and reduces the formation of mPTP.^[92]^ Currently, the uncoupling agents DNP, UCP2, and UCP3 have been shown to increase tissue oxygen consumption, reduce ROS levels in rat models of neurodegenerative disease, and play an influential neuroprotective role.^[47]^ However, the effective dose of the uncoupling agent was close to the toxic dose. Owing to the reduction in ATP production, a large amount of heat is generated during the uncoupling process, causing hyperthermia, denaturation, and inactivation of various enzymes, which cannot meet normal life activities, resulting in cell death and organ failure.^[93–97]^

### 3.2. Inhibition of mPTP formation

Administration of the immunosuppressant cyclosporine A in traumatic brain injury and stroke models binds and inhibits mPTP, enhances mitochondrial function, reduces CNS cell death, and has neuroprotective effects.^[98]^ NIM811 (N-methyl-4-isoleucine cyclosporine) is a non-immunosuppressive cyclosporine A analog that binds Cyp D and blocks the binding site of adenine nucleotide translocons, thereby inhibiting pore opening.^[99]^ At present, there are few studies on the use of NIM811 in the treatment of spinal cord injury, but it can be speculated that NIM811 may play a neuroprotective role after spinal cord injury by reducing mitochondrial damage.

### 3.3. Regulation of energy metabolism and scavenging of RNS/ROS

Pyruvate dehydrogenase (PDH) is a crucial enzyme in acetyl-CoA synthesis.^[100]^ Owing to the lack of PDH and insufficient energy supply after SCI, the introduction of alternative energy sources (e.g., “biofuels”) can alleviate mitochondrial dysfunction after SCI. Acetyl-l-carnitine (ALC) is a component of the inner mitochondrial membrane that readily crosses the blood-brain barrier and provides an acetyl group that assists in synthesizing acetyl-CoA, bypassing the need for PDH.^[9]^ ALC can also increase glutathione (GSH) levels in tissues, thereby enhancing its therapeutic effect.^[101]^ Continued administration of ALC after spinal cord injury reduces neuronal degeneration after spinal cord injury in rats, thereby reducing the number of damaged mitochondria. Improved mitochondrial function by maintaining mitochondrial membrane potential reduces cell death in rats after injury.^[102–104]^

Interventions for mitochondrial oxidative stress include the inhibition of RNS/ROS formation, free radical scavenging, and interruption of LP processes. RNS/ROS formation after spinal cord injury is an explosive process and, therefore, requires immediate intervention in the early stages of injury. Alpha-tocopherol, a naturally occurring form of vitamin E, scavenges lipid peroxides and promotes functional recovery.^[47]^ Melatonin has been shown to reduce Bax migration into the mitochondria and cytochrome c release into the cytoplasm.^[105]^ NAC, a thiol variant of the GSH precursor N-acetylcysteine, was administered 15 to 30 minutes after injury to observe improved mitochondrial bioenergetics, increased GSH concentrations, and improved neurological recovery.^[47]^ U-101033 is a neuronal selective LP inhibitor, and administration of the N-type calcium channel blocker SNX-111 antagonizes LP and Ca^2+^ imbalance, and increases mitochondrial protection. At present, some studies have covalently combined antioxidants with molecular targeting substances to reduce their toxicity, but their efficacy on spinal cord injury is not yet apparent.^[47]^ An increase in the markers of oxidative damage occurs 8 hours after SCI and persists for at least 24 hours.^[106]^ This suggests that the intervention of oxidative stress within 8h after spinal cord injury may achieve the best efficacy.

### 3.4. Mitochondrial fission and fusion

The shape and number of mitochondria can be regulated through fission and fusion. As mitochondria cannot be formed de novo, damaged mitochondria can be replaced or removed through mitochondrial fusion and fission, maintaining normal cellular physiology.^[107]^ The current study found that 3 to 6 hours after spinal cord injury in rats, the number of mitochondria in spinal cord neurons decreased and the shape became swollen, but the opposite results were observed 12 to 24 hours after injury. In the early stage of spinal cord injury (within 3–6 hours), the expression of mitochondrial fusion-related proteins mitochondrial fusion-related proteins (Mfn)1 and Mfn2 increases to 12–24 hours after injury, Mfn1 and Mfn2 decreased, and the expression of fission-related proteins Fis1 and dynamin-related protein 1 significantly increased.^[108]^ At the same time, the mitochondrial membrane potential is reduced, cytochrome c is released, and Caspase-3 is activated, eventually leading to apoptosis.^[107]^ These data suggest that fusion and fission are essential factors in the early and late stages of SCI, respectively. Mdivi-1 is a selective dynamin-related protein 1 inhibitor that increases endogenous antioxidant activity and reduces ROS levels and cytochrome c release. Pretreatment of rats with Mdivi-1 increased local tissue ATP levels and mitochondrial membrane potential stability, and decreased the number of apoptotic cells within 72 hours after spinal cord injury.^[109]^ There is still some controversy about whether to administer Mdivi-1 before or after spinal cord injury and which treatment effect is better deserves further discussion.

### 3.5. Mitochondrial transplantation

Studies have shown that exogenous normal mitochondria can provide enough energy for repair or autophagy of damaged mitochondria, avoiding the release of a series of cytokines into the cytoplasm to trigger apoptosis.^[63]^ There are 3 main methods for mitochondrial transplantation. Microinjection was first used and was mainly used for germ cells. The second is the co-culture of healthy mitochondria with cells. Some studies have found that co-culture of mouse-derived mitochondria with human mesenchymal stem cells can effectively restore cellular respiration. Simultaneously, mouse mitochondria were detected in cells using the fluorescent labeling tracer method.^[110]^ The cell-penetrating peptide Pep-1 binds to exogenous mitochondria and mediates their entry into the cytoplasm. Binding to damaged mitochondria improves ATP production by correcting the membrane potential and promoting mitochondrial function recovery.^[110]^ The third is cell and cell co-culture, which is not significantly different from the mitochondrial cell co-culture. Interestingly, mitochondria only seem to transfer from 1 cell type to another, and there is no reverse transfer. It is speculated that this may be related to different cellular properties.

### 3.6. Regulation of mitochondrial biosynthesis (MB)

MB is the mitochondrial self-regulation of growth and differentiation, controlled by the “master regulator” peroxisome proliferator-activated receptor γ coactivator 1α (PGC-1α).^[111,112]^ PGC-1α interacts and co-activates with a variety of transcription factors to initiate the transcription of nuclear DNA-encoded ETC subunits; therefore, the interaction of drugs to modulate this process can increase MB.^[47]^ For example, the agonism of G protein-coupled serotonin and β-adrenergic receptors can activate the Akt/NOS/cGMP pathway and enhance MB.^[47]^ In addition, resveratrol can enhance MB by activating sirtuin 1 to catalyze the deacetylation of PGC-1α, resulting in improved neurological function.^[9,87]^ Studies have found that the expression of PGC-1α is reduced in rats after spinal cord injury, and lentivirus overexpression (PGC-1a) in neuronal cells effectively reduces neuronal cell death.^[88]^ This suggests that the modulation of MB by increasing PGC-1α levels can potentially improve functional recovery after SCI.^[88]^

### 3.7. Protect mitochondria by inhibiting ferroptosis and cuproptosis

The death of cells via ferroptosis, a form of non-apoptotic regulated cell death discovered in 2012 by Dr Brent R. Stockwell, is iron-dependent.^[113]^ Ferroptosis is characterized by the narrowing of mitochondria, thickening of mitochondrial membranes, and decreased mitochondrial crista.^[114]^ Iron-related functions are disrupted by ionic imbalances between ferrous (Fe2+) and ferric (Fe3+) iron via ROS production, which involves the Fenton and Fenton-like reactions.^[115]^ Iron overload and increased lipid peroxides have been reported in many central nervous system diseases, such as Parkinson’s disease, Alzheimer’s disease, traumatic brain injury, and spinal cord injury.^[41,116,117]^ Iron chelators improve motor symptoms in animal models of Parkinson’s disease and in clinical trials.^[118]^ Ferroptosis is a crucial pathway for dopaminergic neuron death, and the application of ferrostatin-1 can reduce neuronal death in vitro.^[119]^

Tsvetkov discovered a new form of cell death triggered by targeted accumulation of Cu in the mitochondria.^[120]^ Copper (Cu) is required for oxygen metabolism, radical oxygen elimination, and iron (Fe) uptake in eukaryotes. The major copper enzymes present in the mitochondria are cytochrome c oxidase, located in the inner mitochondrial membrane, and copper and zinc superoxide dismutase (SOD1), located in the intermembrane space.^[121]^ Copper is required within the mitochondria to supply the Cu(A) and intramembrane Cu(B) sites of cytochrome oxidase. Physiologically, the mitochondrial matrix is a dynamic copper buffer that efficiently distributes metals to the copper-dependent enzymes. Thus, mitochondria are the first responders to copper homeostasis imbalance. The cytoplasm requires copper to supply Sod1, an important antioxidant substance that maintains the stability of nerve cells.^[122]^ However, similar to ferroptosis, Cu ions also participate in the Fenton reaction and are therefore able to drive the production of harmful hydroxyl radicals.^[123]^ High levels of copper accumulation in the mitochondria in a rat model of Wilson disease associated with copper overload disrupts mitochondrial membrane integrity. Simultaneously, it depletes GSH stores and increases oxidative stress-related damage within organelles.^[124]^ Copper chelators reversed the mitochondrial accumulation of copper in vivo, thereby preserving mitochondrial function and structural integrity.^[125]^ Available data suggest that mitochondria retain a dedicated pool of Cu, which is ultimately used to assemble cytochrome c oxidase and SOD1.^[126]^ Therefore, copper chelators effectively ensure mitochondrial stability and maintain intracellular copper metabolism and apoptosis.

## 4. Combined neuroprotective therapy targeting mitochondria

For neuroprotective therapy of spinal cord injury, attempts to inhibit secondary injury cascades by pharmacologically targeting a single injury mechanism have shown varying degrees of protection in animal models. However, no significant effects were observed in clinical trials. Therefore, early intervention by combining 2 or 3 drugs acting on different processes could theoretically improve neuroprotective efficacy. It avoids using only a single species of instability and potentially offers a wider therapeutic window than a single drug.^[127]^ If the combined benefit of multiple treatments targeting different mitochondrial response processes is demonstrated, this may increase the neuroprotective effect in future clinical trials.

## Acknowledgments

The authors would like to thank the Tianjin Union Medical Center for funding this research. The authors appreciate the valuable support and advice provided by Dr Rusen Zhu and Wei Hu.

## Author contributions

**Conceptualization:** Chengjiang Liu, Ru-Sen Zhu, Yidong Liu, Boyuan Ma.

**Project administration:** Xuanhao Fu, Shunli Kan.

**Writing – original draft:** Wei Hu, Chengjiang Liu, Mengmeng Zhou, Xinyan Zhao.

**Writing – review & editing:** Ru-Sen Zhu.

## References

[1] Eckert MJ Martin MJ. Trauma: spinal cord injury. Surg Clin North Am. 2017;97:1031–45. 28958356 10.1016/j.suc.2017.06.008

[2] McDonald JW Sadowsky C. Spinal-cord injury. Lancet. 2002;359:417–25. 11844532 10.1016/S0140-6736(02)07603-1

[3] Donovan J Kirshblum S. Clinical trials in traumatic spinal cord injury. Neurotherapeutics. 2018;15:654–68. 29736858 10.1007/s13311-018-0632-5 PMC6095794

[4] Dalamagkas K Tsintou M Seifalian AM. Stem cells for spinal cord injuries bearing translational potential. Neural Regen Res. 2018;13:35–42. 29451202 10.4103/1673-5374.224360 PMC5840986

[5] Kjell J Olson L. Rat models of spinal cord injury: from pathology to potential therapies. Dis Model Mech. 2016;9:1125–37. 27736748 10.1242/dmm.025833 PMC5087825

[6] Fakhoury M. Spinal cord injury: overview of experimental approaches used to restore locomotor activity. Rev Neurosci. 2015;26:397–405. 25870961 10.1515/revneuro-2015-0001

[7] Karsy M Hawryluk G. Modern medical management of spinal cord injury. Curr Neurol Neurosci Rep. 2019;19:65. 31363857 10.1007/s11910-019-0984-1

[8] Rabchevsky AG Michael FM Patel SP. Mitochondria focused neurotherapeutics for spinal cord injury. Exp Neurol. 2020;330:113332. 32353464 10.1016/j.expneurol.2020.113332 PMC9164988

[9] Li J Cao F Yin HL. Ferroptosis: past, present and future. Cell Death Dis. 2020;11:88. 32015325 10.1038/s41419-020-2298-2 PMC6997353

[10] Liang C Zhang X Yang M. Recent progress in ferroptosis inducers for cancer therapy. Adv Mater. 2019;31:e1904197. 31595562 10.1002/adma.201904197

[11] McEwen ML Sullivan PG Rabchevsky AG. Targeting mitochondrial function for the treatment of acute spinal cord injury. Neurotherapeutics. 2011;8:168–79. 21360236 10.1007/s13311-011-0031-7 PMC3101832

[12] Fan B Wei Z Yao X. Microenvironment imbalance of spinal cord injury. Cell Transplant. 2018;27:853–66. 29871522 10.1177/0963689718755778 PMC6050904

[13] Kinnally KW Peixoto PM Ryu SY. Is mPTP the gatekeeper for necrosis, apoptosis, or both? Biochim Biophys Acta. 2011;1813:616–22. 20888866 10.1016/j.bbamcr.2010.09.013 PMC3050112

[14] Springer JE Prajapati P Sullivan PG. Targeting the mitochondrial permeability transition pore in traumatic central nervous system injury. Neural Regen Res. 2018;13:1338–41. 30106036 10.4103/1673-5374.235218 PMC6108215

[15] Dumont RJ Okonkwo DO Verma S. Acute spinal cord injury, part I: pathophysiologic mechanisms. Clin Neuropharmacol. 2001;24:254–64. 11586110 10.1097/00002826-200109000-00002

[16] Hu Z Tu J. The roads to mitochondrial dysfunction in a rat model of posttraumatic syringomyelia. Biomed Res Int. 2015;2015:831490. 25685811 10.1155/2015/831490 PMC4309244

[17] Adibhatla RM Hatcher JF. Lipid oxidation and peroxidation in CNS health and disease: from molecular mechanisms to therapeutic opportunities. Antioxid Redox Signal. 2010;12:125–69. 19624272 10.1089/ars.2009.2668

[18] Lee H Zandkarimi F Zhang Y. Energy-stress-mediated AMPK activation inhibits ferroptosis. Nat Cell Biol. 2020;22:225–34. 32029897 10.1038/s41556-020-0461-8 PMC7008777

[19] Hassannia B Vandenabeele P Vanden Berghe T. Targeting ferroptosis to iron out cancer. Cancer Cell. 2019;35:830–49. 31105042 10.1016/j.ccell.2019.04.002

[20] Doll S Freitas FP Shah R. FSP1 is a glutathione-independent ferroptosis suppressor. Nature. 2019;575:693–8. 31634899 10.1038/s41586-019-1707-0

[21] Hao J Li B Duan HQ. Mechanisms underlying the promotion of functional recovery by deferoxamine after spinal cord injury in rats. Neural Regen Res. 2017;12:959–68. 28761430 10.4103/1673-5374.208591 PMC5514872

[22] Ghavami S Shojaei S Yeganeh B. Autophagy and apoptosis dysfunction in neurodegenerative disorders. Prog Neurobiol. 2014;112:24–49. 24211851 10.1016/j.pneurobio.2013.10.004

[23] Sun B Gao Y Lou D. Expression of G-protein-coupled receptor kinase 6 (GRK6) after acute spinal cord injury in adult rat. J Mol Histol. 2013;44:259–70. 23359120 10.1007/s10735-013-9486-7

[24] Hirschhorn T Stockwell BR. The development of the concept of ferroptosis. Free Radic Biol Med. 2019;133:130–43. 30268886 10.1016/j.freeradbiomed.2018.09.043 PMC6368883

[25] Mou Y Wang J Wu J. Ferroptosis, a new form of cell death: opportunities and challenges in cancer. J Hematol Oncol. 2019;12:34. 30925886 10.1186/s13045-019-0720-y PMC6441206

[26] Sun Y Chen P Zhai B. The emerging role of ferroptosis in inflammation. Biomed Pharmacother. 2020;127:110108. 32234642 10.1016/j.biopha.2020.110108

[27] Hall ED Springer JE. Neuroprotection and acute spinal cord injury: a reappraisal. NeuroRx. 2004;1:80–100. 15717009 10.1602/neurorx.1.1.80 PMC534914

[28] Springer JE Visavadiya NP Sullivan PG. Post-injury treatment with NIM811 promotes recovery of function in adult female rats after spinal cord contusion: a dose-response study. J Neurotrauma. 2018;35:492–9. 28967329 10.1089/neu.2017.5167 PMC5793953

[29] Picard M McEwen BS Epel ES. An energetic view of stress: focus on mitochondria. Front Neuroendocrinol. 2018;49:72–85. 29339091 10.1016/j.yfrne.2018.01.001 PMC5964020

[30] Ma Z Xin Z Di W. Melatonin and mitochondrial function during ischemia/reperfusion injury. Cell Mol Life Sci. 2017;74:3989–98. 28795196 10.1007/s00018-017-2618-6 PMC11107672

[31] Pivovarova NB Andrews SB. Calcium-dependent mitochondrial function and dysfunction in neurons. FEBS J. 2010;277:3622–36. 20659161 10.1111/j.1742-4658.2010.07754.x PMC3489481

[32] Sarkar C Zhao Z Aungst S. Impaired autophagy flux is associated with neuronal cell death after traumatic brain injury. Autophagy. 2014;10:2208–22. 25484084 10.4161/15548627.2014.981787 PMC4502690

[33] Sullivan PG Rabchevsky AG Keller JN. Intrinsic differences in brain and spinal cord mitochondria: implication for therapeutic interventions. J Comp Neurol. 2004;474:524–34. 15174070 10.1002/cne.20130

[34] Finsterer J. Clinical therapeutic management of human mitochondrial disorders. Pediatr Neurol. 2020;113:66–74. 33053453 10.1016/j.pediatrneurol.2020.07.004

[35] Hall ED Wang JA Bosken JM. Lipid peroxidation in brain or spinal cord mitochondria after injury. J Bioenerg Biomembr. 2016;48:169–74. 25595872 10.1007/s10863-015-9600-5 PMC4506732

[36] Butterfield DA. Brain lipid peroxidation and Alzheimer disease: synergy between the butterfield and mattson laboratories. Ageing Res Rev. 2020;64:101049. 32205035 10.1016/j.arr.2020.101049 PMC7502429

[37] Bains M Hall ED. Antioxidant therapies in traumatic brain and spinal cord injury. Biochim Biophys Acta. 2012;1822:675–84. 22080976 10.1016/j.bbadis.2011.10.017 PMC4134010

[38] Piastra M Caresta E Massimi L. Lipid peroxidation and antioxidant consumption as early markers of neurosurgery-related brain injury in children. Neurocrit Care. 2020;33:124–31. 31696410 10.1007/s12028-019-00870-w

[39] Anthonymuthu TS Kenny EM Bayir H. Therapies targeting lipid peroxidation in traumatic brain injury. Brain Res. 2016;1640(Pt A):57–76. 26872597 10.1016/j.brainres.2016.02.006 PMC4870119

[40] Wang H Liu C Zhao Y. Mitochondria regulation in ferroptosis. Eur J Cell Biol. 2020;99:151058. 31810634 10.1016/j.ejcb.2019.151058

[41] Zhang Y Sun C Zhao C. Ferroptosis inhibitor SRS 16-86 attenuates ferroptosis and promotes functional recovery in contusion spinal cord injury. Brain Res. 2019;1706:48–57. 30352209 10.1016/j.brainres.2018.10.023

[42] Vaishnav RA Singh IN Miller DM. Lipid peroxidation-derived reactive aldehydes directly and differentially impair spinal cord and brain mitochondrial function. J Neurotrauma. 2010;27:1311–20. 20392143 10.1089/neu.2009.1172 PMC2942874

[43] Christie SD Comeau B Myers T. Duration of lipid peroxidation after acute spinal cord injury in rats and the effect of methylprednisolone. Neurosurg Focus. 2008;25:E5. 10.3171/FOC.2008.25.11.E5 18980479

[44] Liu J Du L. PERK pathway is involved in oxygen-glucose-serum deprivation-induced NF-kB activation via ROS generation in spinal cord astrocytes. Biochem Biophys Res Commun. 2015;467:197–203. 26454173 10.1016/j.bbrc.2015.10.007

[45] Temiz C Solmaz I Tehli O. The effects of splenectomy on lipid peroxidation and neuronal loss in experimental spinal cord ischemia/reperfusion injury. Turk Neurosurg. 2013;23:67–74. 23344870 10.5137/1019-5149.JTN.6825-12.1

[46] Shibata N Nagai R Uchida K. Morphological evidence for lipid peroxidation and protein glycoxidation in spinal cords from sporadic amyotrophic lateral sclerosis patients. Brain Res. 2001;917:97–104. 11602233 10.1016/s0006-8993(01)02926-2

[47] Islam MT. Oxidative stress and mitochondrial dysfunction-linked neurodegenerative disorders. Neurol Res. 2017;39:73–82. 27809706 10.1080/01616412.2016.1251711

[48] Xu T Ding W Ji X. Molecular mechanisms of ferroptosis and its role in cancer therapy. J Cell Mol Med. 2019;23:4900–12. 31232522 10.1111/jcmm.14511 PMC6653007

[49] Weiland A Wang Y Wu W. Ferroptosis and its role in diverse brain diseases. Mol Neurobiol. 2019;56:4880–93. 30406908 10.1007/s12035-018-1403-3 PMC6506411

[50] Wang W Green M Choi JE. CD8(+) T cells regulate tumour ferroptosis during cancer immunotherapy. Nature. 2019;569:270–4. 31043744 10.1038/s41586-019-1170-y PMC6533917

[51] Schapira AHV. Mitochondrial disease. Lancet. 2006;368:70–82. 16815381 10.1016/S0140-6736(06)68970-8

[52] Yu CH Davidson S Harapas CR. TDP-43 triggers mitochondrial DNA release via mPTP to activate cGAS/STING in ALS. Cell. 2020;183:636–49.e18. 33031745 10.1016/j.cell.2020.09.020 PMC7599077

[53] Keeney PM Bennett JP Jr. ALS spinal neurons show varied and reduced mtDNA gene copy numbers and increased mtDNA gene deletions. Mol Neurodegener. 2010;5:21. 20504367 10.1186/1750-1326-5-21 PMC2889994

[54] Conrad M Pratt DA. The chemical basis of ferroptosis. Nat Chem Biol. 2019;15:1137–47. 31740834 10.1038/s41589-019-0408-1

[55] Ye Z Liu W Zhuo Q. Ferroptosis: final destination for cancer? Cell Prolif. 2020;53:e12761. 32100402 10.1111/cpr.12761 PMC7106955

[56] Ubellacker JM Tasdogan A Ramesh V. Lymph protects metastasizing melanoma cells from ferroptosis. Nature. 2020;585:113–8. 32814895 10.1038/s41586-020-2623-z PMC7484468

[57] Simmons EC Scholpa NE Schnellmann RG. Mitochondrial biogenesis as a therapeutic target for traumatic and neurodegenerative CNS diseases. Exp Neurol. 2020;329:113309. 32289315 10.1016/j.expneurol.2020.113309 PMC7735537

[58] Leonard JV Schapira AHV. Mitochondrial respiratory chain disorders II: neurodegenerative disorders and nuclear gene defects. Lancet. 2000;355:389–94. 10665569 10.1016/s0140-6736(99)05226-5

[59] Finsterer J Zarrouk-Mahjoub S. Involvement of the spinal cord in mitochondrial disorders. J Neurosci Rural Pract. 2018;9:245–51. 29725177 10.4103/jnrp.jnrp_446_17 PMC5912032

[60] Genin EC Madji Hounoum B Bannwarth S. Mitochondrial defect in muscle precedes neuromuscular junction degeneration and motor neuron death in CHCHD10(S59L/+) mouse. Acta Neuropathol. 2019;138:123–45. 30874923 10.1007/s00401-019-01988-z

[61] Scholpa NE Schnellmann RG. Mitochondrial-based therapeutics for the treatment of spinal cord injury: mitochondrial biogenesis as a potential pharmacological target. J Pharmacol Exp Ther. 2017;363:303–13. 28935700 10.1124/jpet.117.244806 PMC5676296

[62] Ladd AC Keeney PM Govind MM. Mitochondrial oxidative phosphorylation transcriptome alterations in human amyotrophic lateral sclerosis spinal cord and blood. Neuromolecular Med. 2014;16:714–26. 25081190 10.1007/s12017-014-8321-y

[63] Zhou B Yu P Lin MY. Facilitation of axon regeneration by enhancing mitochondrial transport and rescuing energy deficits. J Cell Biol. 2016;214:103–19. 27268498 10.1083/jcb.201605101 PMC4932375

[64] Korimova A Klusakova I Hradilova-Svizenska I. Mitochondrial damage-associated molecular patterns of injured axons induce outgrowth of Schwann cell processes. Front Cell Neurosci. 2018;12:457. 30542268 10.3389/fncel.2018.00457 PMC6277938

[65] Han Q Xie Y Ordaz JD. Restoring cellular energetics promotes axonal regeneration and functional recovery after spinal cord injury. Cell Metab. 2020;31:623–41.e8. 32130884 10.1016/j.cmet.2020.02.002 PMC7188478

[66] Latunde-Dada GO. Ferroptosis: role of lipid peroxidation, iron and ferritinophagy. Biochim Biophys Acta Gen Subj. 2017;1861:1893–900. 28552631 10.1016/j.bbagen.2017.05.019

[67] Wu J Minikes AM Gao M. Intercellular interaction dictates cancer cell ferroptosis via NF2-YAP signalling. Nature. 2019;572:402–6. 31341276 10.1038/s41586-019-1426-6 PMC6697195

[68] Smith GM Gallo G. The role of mitochondria in axon development and regeneration. Dev Neurobiol. 2018;78:221–37. 29030922 10.1002/dneu.22546 PMC5816701

[69] Fang SY Roan JN Lee JS. Transplantation of viable mitochondria attenuates neurologic injury after spinal cord ischemia. J Thorac Cardiovasc Surg. 2021;161:e337–47. 31866084 10.1016/j.jtcvs.2019.10.151

[70] Sullivan PG Krishnamurthy S Patel SP. Temporal characterization of mitochondrial bioenergetics after spinal cord injury. J Neurotrauma. 2007;24:991–9. 17600515 10.1089/neu.2006.0242

[71] Kubli DA Gustafsson AB. Mitochondria and mitophagy: the yin and yang of cell death control. Circ Res. 2012;111:1208–21. 23065344 10.1161/CIRCRESAHA.112.265819 PMC3538875

[72] Zhang X Yan H Yuan Y. Cerebral ischemia-reperfusion-induced autophagy protects against neuronal injury by mitochondrial clearance. Autophagy. 2013;9:1321–33. 23800795 10.4161/auto.25132

[73] Yuan Y Zhang X Zheng Y. Regulation of mitophagy in ischemic brain injury. Neurosci Bull. 2015;31:395–406. 26219224 10.1007/s12264-015-1544-6 PMC5563715

[74] Eiyama A Okamoto K. PINK1/Parkin-mediated mitophagy in mammalian cells. Curr Opin Cell Biol. 2015;33:95–101. 25697963 10.1016/j.ceb.2015.01.002

[75] Pickrell AM Youle RJ. The roles of PINK1, parkin, and mitochondrial fidelity in Parkinson’s disease. Neuron. 2015;85:257–73. 25611507 10.1016/j.neuron.2014.12.007 PMC4764997

[76] Quinn PMJ Moreira PI Ambrosio AF. PINK1/PARKIN signalling in neurodegeneration and neuroinflammation. Acta Neuropathol Commun. 2020;8:189. 33168089 10.1186/s40478-020-01062-w PMC7654589

[77] Wang XJ Qi L Cheng YF. PINK1 overexpression prevents forskolin-induced tau hyperphosphorylation and oxidative stress in a rat model of Alzheimer’s disease. Acta Pharmacol Sin. 2021;43:1916–27. 34893682 10.1038/s41401-021-00810-5 PMC9343460

[78] Wen S Wang L Wang T. Puerarin alleviates cadmium-induced mitochondrial mass decrease by inhibiting PINK1-Parkin and Nix-mediated mitophagy in rat cortical neurons. Ecotoxicol Environ Saf. 2021;230:113127. 34979308 10.1016/j.ecoenv.2021.113127

[79] Vives-Bauza C Zhou C Huang Y. PINK1-dependent recruitment of Parkin to mitochondria in mitophagy. Proc Natl Acad Sci USA. 2010;107:378–83. 19966284 10.1073/pnas.0911187107 PMC2806779

[80] Gu C Li L Huang Y. Salidroside ameliorates mitochondria-dependent neuronal apoptosis after spinal cord ischemia-reperfusion injury partially through inhibiting oxidative stress and promoting mitophagy. Oxid Med Cell Longev. 2020;2020:3549704. 32774670 10.1155/2020/3549704 PMC7396093

[81] Mao Y Du J Chen X. Maltol promotes mitophagy and inhibits oxidative stress via the Nrf2/PINK1/Parkin pathway after spinal cord injury. Oxid Med Cell Longev. 2022;2022:1337630. 35154562 10.1155/2022/1337630 PMC8826207

[82] Huang EJ Reichardt LF. Neurotrophins: roles in neuronal development and function. Annu Rev Neurosci. 2001;24:677–736. 11520916 10.1146/annurev.neuro.24.1.677 PMC2758233

[83] Chay W Kirshblum S. Predicting outcomes after spinal cord injury. Phys Med Rehabil Clin N Am. 2020;31:331–43. 32624098 10.1016/j.pmr.2020.03.003

[84] Totoiu MO Keirstead HS. Spinal cord injury is accompanied by chronic progressive demyelination. J Comp Neurol. 2005;486:373–83. 15846782 10.1002/cne.20517

[85] Stein DM Sheth KN. Management of acute spinal cord injury. Continuum (Minneap Minn). 2015;21:159–87. 25651224 10.1212/01.CON.0000461091.09736.0c

[86] Fouad K Schnell L Bunge MB. Combining Schwann cell bridges and olfactory-ensheathing glia grafts with chondroitinase promotes locomotor recovery after complete transection of the spinal cord. J Neurosci. 2005;25:1169–78. 15689553 10.1523/JNEUROSCI.3562-04.2005 PMC6725952

[87] Yu F Zhang Q Liu H. Dynamic O-GlcNAcylation coordinates ferritinophagy and mitophagy to activate ferroptosis. Cell Discov. 2022;8:40. 35504898 10.1038/s41421-022-00390-6 PMC9065108

[88] Su Y Zhao B Zhou L. Ferroptosis, a novel pharmacological mechanism of anti-cancer drugs. Cancer Lett. 2020;483:127–36. 32067993 10.1016/j.canlet.2020.02.015

[89] Chen X Yu C Kang R. Iron metabolism in ferroptosis. Front Cell Dev Biol. 2020;8:590226. 33117818 10.3389/fcell.2020.590226 PMC7575751

[90] Gollihue JL Patel SP Rabchevsky AG. Mitochondrial transplantation strategies as potential therapeutics for central nervous system trauma. Neural Regen Res. 2018;13:194–7. 29557359 10.4103/1673-5374.226382 PMC5879881

[91] Wei X Yi X Zhu XH. Posttranslational modifications in ferroptosis. Oxid Med Cell Longev. 2020;2020:8832043. 33294126 10.1155/2020/8832043 PMC7718049

[92] Badgley MA Kremer DM Maurer HC. Cysteine depletion induces pancreatic tumor ferroptosis in mice. Science. 2020;368:85–9. 32241947 10.1126/science.aaw9872 PMC7681911

[93] Jastroch M. Uncoupling protein 1 controls reactive oxygen species in brown adipose tissue. Proc Natl Acad Sci USA. 2017;114:7744–6. 28710335 10.1073/pnas.1709064114 PMC5544340

[94] Nagoshi N Tsuji O Nakamura M. Cell therapy for spinal cord injury using induced pluripotent stem cells. Regen Ther. 2019;11:75–80. 31245451 10.1016/j.reth.2019.05.006 PMC6581851

[95] Szalowska E Pronk TE Peijnenburg AA. Cyclosporin A induced toxicity in mouse liver slices is only slightly aggravated by Fxr-deficiency and co-occurs with upregulation of pro-inflammatory genes and downregulation of genes involved in mitochondrial functions. BMC Genomics. 2015;16:822. 26482353 10.1186/s12864-015-2054-7 PMC4617705

[96] Readnower RD Pandya JD McEwen ML. Post-injury administration of the mitochondrial permeability transition pore inhibitor, NIM811, is neuroprotective and improves cognition after traumatic brain injury in rats. J Neurotrauma. 2011;28:1845–53. 21875332 10.1089/neu.2011.1755 PMC3172877

[97] Gollihue JL Rabchevsky AG. Prospects for therapeutic mitochondrial transplantation. Mitochondrion. 2017;35:70–9. 28533168 10.1016/j.mito.2017.05.007 PMC5518605

[98] Karalija A Novikova LN Kingham PJ. Neuroprotective effects of N-acetyl-cysteine and acetyl-L-carnitine after spinal cord injury in adult rats. PLoS One. 2012;7:e41086. 22815926 10.1371/journal.pone.0041086 PMC3398872

[99] Patel SP Sullivan PG Lyttle TS. Acetyl-L-carnitine treatment following spinal cord injury improves mitochondrial function correlated with remarkable tissue sparing and functional recovery. Neuroscience. 2012;210:296–307. 22445934 10.1016/j.neuroscience.2012.03.006 PMC3358433

[100] Lu B Chen XB Ying MD. The role of ferroptosis in cancer development and treatment response. Front Pharmacol. 2017;8:992. 29375387 10.3389/fphar.2017.00992 PMC5770584

[101] Feng H Schorpp K Jin J. Transferrin receptor is a specific ferroptosis marker. Cell Rep. 2020;30:3411–23.e7. 32160546 10.1016/j.celrep.2020.02.049 PMC7172030

[102] Radogna F Albertini MC De Nicola M. Melatonin promotes Bax sequestration to mitochondria reducing cell susceptibility to apoptosis via the lipoxygenase metabolite 5-hydroxyeicosatetraenoic acid. Mitochondrion. 2015;21:113–21. 25702644 10.1016/j.mito.2015.02.003

[103] Jia ZQ Li G Zhang ZY. Time representation of mitochondrial morphology and function after acute spinal cord injury. Neural Regen Res. 2016;11:137–43. 26981103 10.4103/1673-5374.175061 PMC4774207

[104] Ranieri M Del Bo R Bordoni A. Optic atrophy plus phenotype due to mutations in the OPA1 gene: two more Italian families. J Neurol Sci. 2012;315:146–9. 22197506 10.1016/j.jns.2011.12.002 PMC3315002

[105] Cao Y Lv G Wang YS. Mitochondrial fusion and fission after spinal sacord injury in rats. Brain Res. 2013;1522:59–66. 23727406 10.1016/j.brainres.2013.05.033

[106] Li G Jia Z Cao Y. Mitochondrial division inhibitor 1 ameliorates mitochondrial injury, apoptosis, and motor dysfunction after acute spinal cord injury in rats. Neurochem Res. 2015;40:1379–92. 25968480 10.1007/s11064-015-1604-3

[107] Chang JC Liu KH Li YC. Functional recovery of human cells harbouring the mitochondrial DNA mutation MERRF A8344G via peptide-mediated mitochondrial delivery. Neurosignals. 2013;21:160–73. 23006856 10.1159/000341981

[108] Song W Song Y Kincaid B. Mutant SOD1G93A triggers mitochondrial fragmentation in spinal cord motor neurons: neuroprotection by SIRT3 and PGC-1alpha. Neurobiol Dis. 2013;51:72–81. 22819776 10.1016/j.nbd.2012.07.004 PMC3992938

[109] Hu J Lang Y Zhang T. Lentivirus-mediated PGC-1alpha overexpression protects against traumatic spinal cord injury in rats. Neuroscience. 2016;328:40–9. 27132229 10.1016/j.neuroscience.2016.04.031

[110] Lu P Han D Zhu K. Effects of Sirtuin 1 on microglia in spinal cord injury: involvement of Wnt/beta-catenin signaling pathway. Neuroreport. 2019;30:867–74. 31373965 10.1097/WNR.0000000000001293

[111] Hu J Lang Y Cao Y. The neuroprotective effect of tetramethylpyrazine against contusive spinal cord injury by activating PGC-1alpha in rats. Neurochem Res. 2015;40:1393–401. 25981953 10.1007/s11064-015-1606-1 PMC4493940

[112] Yousefifard M Rahimi-Movaghar V Nasirinezhad F. Neural stem/progenitor cell transplantation for spinal cord injury treatment; a systematic review and meta-analysis. Neuroscience. 2016;322:377–97. 26917272 10.1016/j.neuroscience.2016.02.034

[113] Dixon SJ Lemberg KM Lamprecht MR. Ferroptosis: an iron-dependent form of nonapoptotic cell death. Cell. 2012;149:1060–72. 22632970 10.1016/j.cell.2012.03.042 PMC3367386

[114] Ma D Jiang P Jiang Y. Effects of lipid peroxidation-mediated ferroptosis on severe acute pancreatitis-induced intestinal barrier injury and bacterial translocation. Oxid Med Cell Longev. 2021;2021:6644576. 34257815 10.1155/2021/6644576 PMC8245223

[115] Kletetschka G Bazala R Takac M. Magnetic domains oscillation in the brain with neurodegenerative disease. Sci Rep. 2021;11:714. 33436793 10.1038/s41598-020-80212-5 PMC7804002

[116] Do Van B Gouel F Jonneaux A. Ferroptosis, a newly characterized form of cell death in Parkinson’s disease that is regulated by PKC. Neurobiol Dis. 2016;94:169–78. 27189756 10.1016/j.nbd.2016.05.011

[117] Kenny EM Fidan E Yang Q. Ferroptosis contributes to neuronal death and functional outcome after traumatic brain injury. Crit Care Med. 2019;47:410–8. 30531185 10.1097/CCM.0000000000003555 PMC6449247

[118] Shukla JJ Stefanova N Bush AI. Therapeutic potential of iron modulating drugs in a mouse model of multiple system atrophy. Neurobiol Dis. 2021;159:105509. 34537326 10.1016/j.nbd.2021.105509

[119] Kabiraj P Valenzuela CA Marin JE. The neuroprotective role of ferrostatin-1 under rotenone-induced oxidative stress in dopaminergic neuroblastoma cells. Protein J. 2015;34:349–58. 26385697 10.1007/s10930-015-9629-7

[120] Tsvetkov P Detappe A Cai K. Mitochondrial metabolism promotes adaptation to proteotoxic stress. Nat Chem Biol. 2019;15:681–9. 31133756 10.1038/s41589-019-0291-9 PMC8183600

[121] Baker ZN Cobine PA Leary SC. The mitochondrion: a central architect of copper homeostasis. Metallomics. 2017;9:1501–12. 28952650 10.1039/c7mt00221a PMC5688007

[122] Leary SC Winge DR Cobine PA. “Pulling the plug” on cellular copper: the role of mitochondria in copper export. Biochim Biophys Acta. 2009;1793:146–53. 18522804 10.1016/j.bbamcr.2008.05.002 PMC4021392

[123] Robinson NJ Winge DR. Copper metallochaperones. Annu Rev Biochem. 2010;79:537–62. 20205585 10.1146/annurev-biochem-030409-143539 PMC3986808

[124] Zischka H Lichtmannegger J. Pathological mitochondrial copper overload in livers of Wilson’s disease patients and related animal models. Ann N Y Acad Sci. 2014;1315:6–15. 24517326 10.1111/nyas.12347

[125] Zischka H Lichtmannegger J Schmitt S. Liver mitochondrial membrane crosslinking and destruction in a rat model of Wilson disease. J Clin Invest. 2011;121:1508–18. 21364284 10.1172/JCI45401 PMC3068979

[126] Cobine PA Moore SA Leary SC. Getting out what you put in: copper in mitochondria and its impacts on human disease. Biochim Biophys Acta Mol Cell Res. 2021;1868:118867. 32979421 10.1016/j.bbamcr.2020.118867 PMC7680424

[127] Kabu S Gao Y Kwon BK Labhasetwar V. Drug delivery, cell-based therapies, and tissue engineering approaches for spinal cord injury. J Control Release. 2015;219:141–54. 26343846 10.1016/j.jconrel.2015.08.060 PMC4656085

